# “Walk for Life”: A Feasibility Randomised Controlled Trial of Guolin Qigong for Fatigue, Sleep Disturbance and Depression Symptom Cluster in Cancer Survivors

**DOI:** 10.1177/15347354261442682

**Published:** 2026-05-04

**Authors:** Sara L. K. Low, Gwo Fuang Ho, Bingkai Liu, Eng-Siew Koh, Kingsley Agho, Yutong Fei, Xiaoshu Zhu

**Affiliations:** 1 Western Sydney University, NSW, Australia; 2 Australia-China Chinese Medicine Centre (An International Collaboration Between Western Sydney University and Beijing University of Chinese Medicine), Australia; 3 Universiti Malaya, Kuala Lumpur, Malaysia; 4 Pitié-Salpêtrière Hospital, Paris, France; 5 Liverpool Hospital, Liverpool, NSW, Australia; 6 University of New South Wales, Sydney, NSW, Australia; 7 Beijing University of Chinese Medicine, China

**Keywords:** Qigong, cancer survivors, fatigue, sleep disturbances, depression, urine cortisol, mind- body exercise

## Abstract

**Purpose:**

To evaluate the feasibility and the preliminary estimate of effect of a 12-week Guolin Qigong (GQ) intervention, a mind-body exercise, on the fatigue-sleep disturbance-depression symptoms among breast, lung and colon cancer survivors.

**Methods:**

Forty-one breast, lung, and colon cancer survivors with moderate to severe fatigue as measured by the Brief Fatigue Index were randomised to a GQ intervention (n = 21) or a waitlist control (n = 20). Outcome measures were assessed at baseline, 6, 12 weeks (post-intervention), and 16 weeks (4 weeks post-intervention). The primary feasibility was assessed using recruitment, retention, class attendance, home practice, safety and quantum of missing data. Secondary outcomes of symptom differences across timepoints in fatigue, sleep, and depression were assessed using a Generalised Estimating Equations (GEE) model with a Gaussian family, independent link, exchangeable correlation structure, and robust variance estimation.

**Results:**

Forty-one cancer survivors were successfully recruited with a recruitment rate of 72%. Retention was 95% (GQ) and 80% (control) at Week-12 and Week-16. GQ intervention class attendance was 86% for the 12-weeks duration with home practice averaging 5 day/week and 394 minutes/week. Missing data were less than 10%. There were no intervention-related serious adverse events. From baseline to Week 12 or Week 16, the average decreases in fatigue, sleep quality, and depression were 0.66, 0.11, and 1.79 respectively, after adjusting for gender and cancer-type imbalances.

**Conclusion:**

GQ was feasible and safe for cancer survivors with fatigue-sleep disturbance-depression symptoms, within the defined small sample size. A larger randomised trial is needed for statistical validation.

## Introduction

Cancer-related fatigue is a complex interplay of physical, emotional, and cognitive responses to the stressors related to diagnosis and treatment. It is intricately linked with factors such as sleep disturbances, depression, anaemia, and inflammation affecting cancer survivors from diagnosis, treatment and into survivorship.^1^ Cancer related fatigue, sleep disturbance, and depression are the most commonly co-occurring symptoms defined as a cluster.^2-6^ Some of these associations are potentially linked to the underlying inflammatory changes such as cortisol levels^7^ most associated with fatigue.^8-10^ Cancer survivors experiencing such symptoms report poorer quality of life,^2,5,11-13^ reduced physical and cognitive function, and reduced overall survival than those with few or no symptoms.^14^ These symptoms lead to compounded distress, reduced social functioning, and prolonged recovery periods.^15^ Although improvement in the fatigue, sleep disturbance, and depression (FSD) symptoms was associated with increase in quality of life in cancer survivors,^11,12^ there are no effective pharmaceutical agents without significant side effect profiles for managing these symptoms. This highlights a critical gap in symptom management strategies and underscores the need for integrative and non-pharmacological interventions to effectively address these interconnected symptoms and improve overall well-being of cancer survivors.

Qigong, rooted in Traditional Chinese Medicine (TCM), encompasses various forms of mind-body practice that integrate gentle movements, coordinated breathing, and focussed intention, all grounded in the recognition and cultivation of Qi (often translated as vital energy).^16,17^ It is generally defined as “one type of low-level aerobic, gentle healthcare and self-healing exercises,”^18^ practiced to enhance physical health, mental clarity, and spiritual well-being.

Evidence suggests that Qigong offers multidimensional benefits, including physical function, psychological health, and overall quality of life across a wide range of conditions and multimorbidity.^19,20^ Its therapeutic effects are thought to be mediated, in part, through the inhibition of proinflammatory cytokine production^21,22^ and reduced inflammation,^23,24^ which is closely associated with fatigue, sleep disturbance, and depression.^25,26^ Evidence from randomised controlled trial (RCT)^5^ and systematic reviews^27-29^ indicates that Qigong can effectively alleviate these fatigue, sleep disturbance, and depression symptoms in cancer survivors. For example, Tai Chi, a form of Qigong, demonstrated large effect sizes in reducing fatigue, improving sleep, and alleviating depression in breast cancer survivors, underscoring the interplay of these symptoms and their collective impact on wellbeing.^5^ Cancer survivors reported it to be generally convenient, energy saving, and low-intensity for managing fatigue-sleep disturbance-depression symptoms.^5^ Compared with conventional therapies, Qigong offers several advantages: it takes a holistic approach, fosters self-empowerment,^30^ has not been linked to serious adverse effects when practiced under proper guidance,^31-33^ and may be more cost-effective, as it can be practiced independently without ongoing expenses once learned.^30,34,35^

Among the many forms and styles of Qigong, Guolin Qigong (GQ) is distinctive in that it was specifically developed for rehabilitation of individuals with cancer. Unlike many forms of Qigong that emphasise static postures or complex sequences such as Tai Chi, GQ integrates moderate walking with coordinated arm movements, trunk rotations, and specialised breathing techniques in a gentle, continuous and repetitive manner. A defining feature of this intervention its exceptional adaptability, allowing modifications in activity speed, duration, and intensity according to individual health conditions. This patient-centred approach makes it particularly suitable for cancer survivors, especially those with advanced stage or functional limitations who may find other forms of physical activity challenging.^36^ Furthermore, the unique breathing technique used during GQ supports oxygen–carbon dioxide homoeostasis by enhancing oxygen inhalation, which may contribute to cancer control and overall survival.^37^

As we understand, although GQ is widely practiced among survivors in China, Malaysia, and other countries, its effects remain largely unexplored in the scientific literature. To the best of our knowledge, no rigorous studies have specifically examined the impact of GQ on managing FSD symptoms and its correlation with cortisol level in breast, lung and colon cancer survivors. This gap highlighted the need for further investigation. Therefore, this study was designed to explore the feasibility and the preliminary estimate of effects of a 12-week GQ intervention on FSD, and cortisol level in cancer survivors.

## Methods

### Study Design

We conducted a randomised-controlled trial (RCT) to compare a 12-week GQ intervention with a usual care waitlist control group of 41 eligible breast, lung, and colon cancer survivors. This study has been registered with ANZCTR (ACTRN12622000688785p), was approved by Medical Research Ethic Committee of University Malaya Medical Centre (MREC ID NO: 2022323-11092) and recognised by Western Sydney University Human Research Ethics Committee (RH15124).

In addition, we also interviewed trial participants to explore their perceptions and experiences of the intervention. The qualitative findings will be reported in a subsequent paper, while the current study focuses on feasibility and clinical outcomes. Insights from this trial will inform the design, hypotheses, and methodology for a future larger RCT to evaluate the impact of GQ on health outcomes in this population The detailed study protocol has been published elsewhere.^30^ A brief summary of the RCT design is provided below.

### Study Setting, Sample, Randomisation and Allocation Concealment

The trial was conducted at University Malaya Medical Centre (UMMC) in Kuala Lumpur, Malaysia. 41 breast, lung and colon cancer survivors reporting moderate to severe fatigue with Brief Fatigue Inventory (BFI) score of ≥3 were recruited from October 2022-July 2023.

Participants were recruited by referrals from UMMC or online and print advertisements such as posting recruitment in UMMC, cancer support groups, GQ Association and GQ practice stations around Klang Valley, and social media platforms including × Corp (formerly known as Twitter) and Facebook.

After screening a by Physician/ Oncologist and informed consent, participants were randomised to either the GQ group or waitlist control group with the random sequence generated by an online computerised randomisation system using minimisation method^38-40^ by an independent research assistant.

Inclusion criteria:

(1) Age ≥18 years old;
(2) Cancer survivor diagnosed with breast, lung and colorectal cancer (a cancer survivor is defined as an individual diagnosed with cancer through the balance of life, according to National Cancer Institute^41^);
(3) Able to read and answer questionnaires in English and/or Bahasa Malaysia language;
(4) Suffering from moderate to severe fatigue as assessed by the simple fatigue scale, with severity score of 3 and above;
(5) Able to use smart phones and the WeChat or WhatsApp application;
(6) Life expectancy of more than 3 months;
(7) Able to give informed consent;
(8) Available and willing to comply with the study requirements.

Exclusion criteria:

(1) Cardiopulmonary disease, nerve, muscle, or joint disease, or other malignant tumours affecting movement;
(2) Mental illness or serious cognitive impairment and defects in language that significantly impairs communication;
(3) Post-operative heart, cerebral vessel, or other serious complications;
(4) Neurological degenerative disease (eg, dementia), reduced cognitive capacity in any way that would affect ability to understand trial procedures and give informed consent;
(5) Patients who are not able to walk;
(6) Other medical conditions which would preclude study intervention or make study participation unsafe such as severe chronic heart failure.

### Sample Size

The initial sample size for this study has been described elsewhere^30^ and was informed by statistical power considerations but was subsequently revised to 80% to ensure appropriateness for a feasibility design. The selected sample size is intended to estimate key feasibility parameters, including recruitment, retention, adherence, and data completeness, and to provide preliminary estimates of variability in clinical outcomes to inform a future fully powered trial. It will also support assessment of intervention acceptability and implementation processes. The study is not powered for hypothesis testing, and outcome analyses will be exploratory and descriptive.

### Study Intervention

#### GQ Intervention

Detailed procedures were outlined in the published study protocol.^30^ The GQ group attended a 12-week GQ intervention, comprised of twice-weekly 2-hour supervised in-person classes for the first 2 weeks, followed by once-weekly classes for the remaining 10 weeks. Participants were requested to practice a minimum of 60 minutes daily home practice adjusted to their health and schedule.^36^ Participants were also supported with standardised instructional materials (class notes and video links) to promote and support home practice.

The GQ intervention was delivered by 2 instructors who were long-term cancer survivors with more than 20 years of experience teaching GQ.

In brief, the classes consisted of smooth and relaxed walking and arm movements with co-ordinated breathing with the goal of relaxing the mind and body. The current study included the following 3 main exercises of GQ:

1. Natural Wind Breathing Walking Method
2. Step Tap Method
3. Ascending, Descending, Opening and Closing Method

Each main exercise can be practised separately, accompanied by the Preparatory Relaxation Exercise (gentle breathing and meditation, opening and closing of Dantian; 4 minutes) at the beginning and followed by the Concluding Exercise (breathing exercise, opening, and closing of the Dantian, and self-massage; 3-6 minutes) at the end.

#### Usual Care Waitlist Control

The waitlist control group continued with their usual care routines and exercises throughout the study period. They were asked to refrain from any other Qigong exercises during the time to minimise potential confounding effects. Participants were not restricted from participating in other cancer support care activities (eg, cancer support workshops or exercise programmes). Information regarding engagement in such activities were obtained through voluntary self-report during study communication and follow-up assessments. Upon completion of the 16-week GQ programme of their cohort, participants in the waitlist control were offered to attend the same GQ training programme.

Due to the visible nature of the GQ intervention, blinding of participants to group allocation was not feasible. To maintain study integrity, intervention instructors were blinded to participants’ allocation status as they were requested to deliver intervention or training without knowledge of group allocation. To mitigate potential bias, standardised instructions protocol were implemented to minimise expectancy effects, and outcome data were collected and cross-verified by independent research assistants.

#### Reporting

Our reporting follows the Consolidated Standards of Reporting Trials (CONSORT) guidelines for pilot studies,^42^ with a primary outcome on evaluating feasibility and secondary outcomes on fatigue, sleep disturbance, and depressive symptoms framed as exploratory and not interpreted as confirmatory efficacy findings, but as a basis to inform the design, outcome selection, and sample size estimation of a future fully powered trial.

### Outcome Measures

#### Primary Feasibility Outcomes

The feasibility of GQ intervention was assessed based on recruitment rate, retention rate, class attendance, home practice adherence, safety and percentage of missing data. Recruitment rate was calculated as the percentage of eligible individuals who consented to participate in the trial. Retention rate was the percentage of enrolled participants who completed the trial and the outcome assessment at Week 12 and Week 16. Class attendance was the percentage of the scheduled number of training classes completed by participants with attendance. Home practice adherence was the number of days and minutes of home practice per week based on practice diaries recorded by participants. Feasibility was assessed using pre-determined cut-off values, of 80% to 85% for retention and 75% to 85% for class attendance.^30^ These thresholds were deemed clinically relevant as they exceeded those reported in similar Qigong studies for cancer survivors, which had retention rates of 60% to 75% and class attendance rates of 54% to 61%.^43-45^

Safety was monitored and assessed based on total number of Adverse Events and the presence of any Serious Adverse Event.^46^ During the 12 weeks of GQ intervention, detailed changes in symptoms and new health events were recorded in the practice diary by the participants.

An adverse event was defined as any unfavourable and unintended sign, symptom, or disease temporally associated with the use of a medical treatment or procedure that may or may not be considered related to the medical treatment or procedure^47^ during the trial. Adverse events were classed as either unrelated GQ adverse events (occurred during the trial but reported as being unrelated to GQ) or GQ-related adverse events (events that occurred during or as a direct result of GQ). Adverse events were categorised by one author (SL) based on severity using the Common Terminology Criteria for Adverse Event (version 5).^47^ Safety was defined as no occurrence of serious adverse events related to the GQ intervention.

During the 12 weeks GQ intervention, participants signed in the attendance sheet to record class attendance. Any dropouts would be followed up and recorded. GQ home practice frequency, duration and any adverse events were recorded throughout the intervention period from Week-1 to Week-12 in the practice diary by the participants. We collected data at baseline (Week-1), mid-intervention (Week-6), post-intervention (Week-12) and followed up on missing data to determine the underlying reasons.

#### Secondary Outcomes – Preliminary Estimate of Effects

1. *Fatigue - The Brief Fatigue Inventory (BFI) was used to* assess fatigue severity in participants.^48^ The BFI is a validated 9-item instrument with items 1 to 3 rating participants’ worst, usual, and current fatigue during the past 24 hours. The remaining 6 items rate the level at which participants’ fatigue interfered with certain functions such as daily activity, work, walking ability, normal work, relations with others, and enjoyment in life.^48^
2. *Sleep disturbance– The Pittsburgh Sleep Quality Index (PSQI)* was used to track participants’ self-reported quality of sleep. The 19-item validated PSQI measures 7 aspects of sleep quality and disturbances; subjective sleep quality, sleep latency, duration, efficiency, disturbances, use of sleep medication, and daytime sleep dysfunction.^49-51^
3. *Depression – The Centre for Epidemiologic Studies Depression Scale (CES-D)* was used to measure depression symptoms. The CES-D is a 20-item validated scale with a maximum score of 60.^52^ Participants with scores ≥16 on the CES-D are considered as having “depressed symptoms.” The cut off score of 12 has been validated with DSM-III criteria for clinical depression.^52,53^

In this study, fatigue, sleep disturbance, and depressive symptoms were conceptualised as an interrelated symptom cluster based on their frequent co-occurrence and shared mechanisms in cancer survivorship. However, as there is currently no validated global instrument specifically designed to assess the fatigue–sleep–depression (FSD) symptoms as a cluster, outcomes were analysed using the validated, symptom-specific patient-reported outcome measures Brief Fatigue Index, Pittsburgh Sleep Quality Index and The Centre for Epidemiologic Studies Depression Scales. Although these tools assess individual symptom domains, each captures a global appraisal of symptom burden within its respective domain, and together they provide a multidimensional assessment of the overall symptoms experience. This approach allowed us to remain conceptually aligned with the symptom cluster framework while using psychometrically robust instruments.

Secondary patient-reported-outcomes on fatigue, sleep disturbance, and depression were measured at baseline (Week-1), mid-intervention (Week-6), post-intervention (Week-12), and 4 weeks after completion of intervention (Week-16).

4. *Cortisol biomarker* – The 24-hour urine cortisol test was included as an exploratory measure to evaluate the effect of the GQ intervention on overall cortisol secretion. By assessing urinary-free cortisol (UFC) across a complete 24-hour collection period, the test provides an integrated estimate of adrenal output over the circadian cycle, offering a more stable indicator of cortisol production than single-time-point measures.^54,55^ This allowed the study to identify potential trends in adrenocortical activity that warrant further investigation.

Urine samples (urine cortisol test) to assess 24-hour urinary cortisol were collected a day prior to enrolment date and post-intervention (Week-12).

5. Other data collection – We collected other baseline information on sociodemographic and cancer history, including cancer type, cancer treatment, current medication, and cancer-related side effects at baseline (Week-1).

All outcomes’ data were collected by an independent research assistant to minimise potential bias.

### Data Analysis

#### Feasibility and Safety

We used descriptive statistics to report study feasibility outcomes of (1) Recruitment (2) Retention (3) Adherence: Class attendance and GQ home practice (days/week) and (minutes/week) across the 12 weeks.

Safety was established through the number of adverse events reported according to relatedness and severity.

#### Preliminary Estimate of Effects

This study was a predetermined exploratory comparison and, as such, was not designed with sufficient power to detect differences between groups. Consequently, in accordance with guidelines for pilot studies, the analyses emphasised effect sizes and confidence intervals to guide the design of future trials, rather than focussing on efficacy and statistical significance.^42,56,57^

We used the intention-to-treat principle for analysis. Continuous data was summarised using descriptive statistics, including mean and standard deviation (SD), and categorical data were summarised as counts and percentages of each category. The 2 groups (intervention and waitlist control) were compared using the appropriate *t*-test, *χ*2 test, or Fisher exact test. Generalised estimating equations (GEE) model with a Gaussian family, independent link, exchangeable correlation structure, and robust variance estimation was used to examine the effect of intervention on BFI, PSQI, and CES-D scores. All analyses were performed using Stata version 17.0 (Stata Corp, College Station, TX, USA), and all statistical tests were 2-tailed, with a *P*-value <.05 denoting statistical significance.

## Results

### Basic Demographic Data (Study Population)

The baseline demographic characteristics (Table 1) of the study arms were comparable, with no significant differences observed. The proportion of females by intervention and control was similar (control: 19; intervention: 19 for females). Among the participants, 44% were aged 40 to 55 years (control: 19.5%; intervention: 24.4%), and 32% were aged 56 to 64 (control: 12.2%; intervention: 19.5%). About 78% of the participants had a history of breast cancer (control: 36.6%; intervention: 41.5%); other types included colon and lung cancer. The mean 24-hour urine cortisol collection was 218.9 in the control group and 83.1 in the intervention. The mean BFI, PSQI, and CES-D were 5.2, 7.4, and 13.2 in the control group and 5.5, 8.3, and 14.2 in the intervention group, respectively. The cancer stages between intervention and control were almost equally distributed for the 4 stages (see Table 1).

**Table 1.** Characteristics of the Patients by Intervention and Control (n = 41).

Variable	Control (n = 20)	Intervention (n = 21)	*P* value
Gender			
Female	19 (90.5)	19 (92.7)	.578
Male	1 (9.5)	2 (7.3)	
Age (years)			
30-39	1 (5.0)	0 (0.0)	.409
40-55	8 (40.0)	10 (47.6)	
56-64	5 (25.0)	8 (38.1)	
≥65	6 (30.0)	3 (14.3)	
Primary cancer type			
Breast	15 (75.0)	17 (81.0)	.805
Colon	3 (15.0)	3 (14.3)	
Lung	2 (10.0)	1 (4.7)	
Cancer stage			
I-III	14 (70.0)	15 (71.4)	.92
IV	6 (30.0)	6 (28.6)	
24-h urine cortisol (mean ± sd)	218.9 ± 357.9	83.1 ± 35.4	.091
Brief fatigue inventory (BFI, mean ± sd)	5.2 ± 1.6	5.5 ± 1.8	.577
Pittsburgh sleep quality index (PSQI, mean ± sd)	7.4 ± 3.3	8.3 ± 3.6	.368
CES-depression (mean ± sd)	13.2 ± 11.2	14.2 ± 11.5	.771

Abbreviations: Sd: standard deviation.

### Primary Feasibility Results

#### Recruitment and Retention

**Figure 1.**
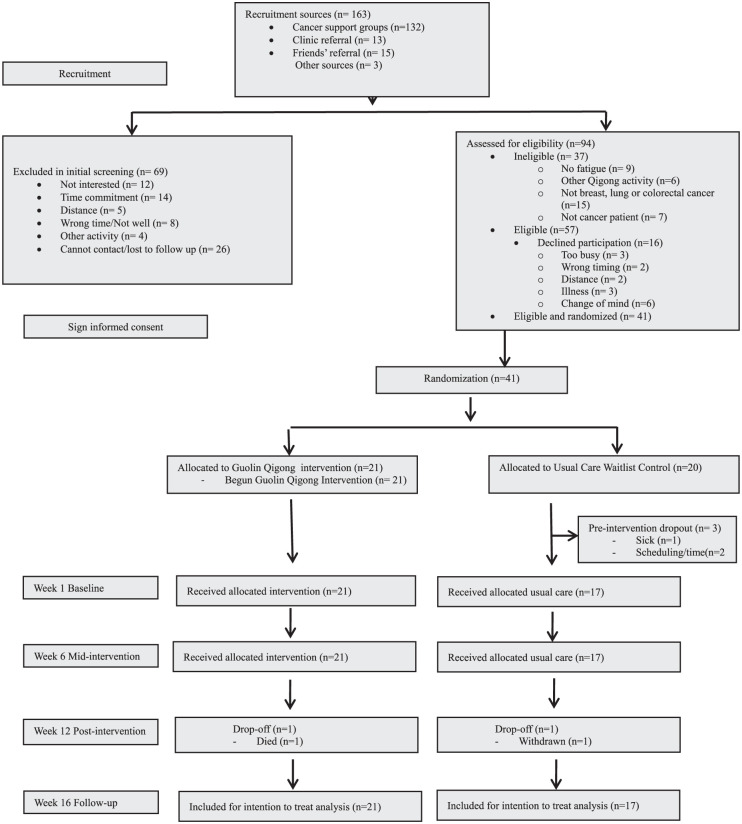
CONSORT diagram of participant recruitment and flow.

The study received 163 inquiries and 69 individuals were exclude during initial screening assessment. Among the 94 assessed for eligibility, 37 were ineligible, and 16 declined participation, resulting in 41 eligible participants were enrolled and randomised into the study (n = 21 GQ, n = 20 waitlist control). Figure 1 shows the CONSORT diagram of flow.

81% of the enquiries were received from cancer support groups (n = 132), followed by friend’s referrals (n = 15,), clinical referrals (n = 13), and other sources (n = 3).

Of the 69 individuals excluded during initial screening, reasons for exclusion were lack of interest (n = 12), time commitment (n = 14), distance (n = 5), scheduling conflicts or illness (n = 8), other activities (n = 4), and loss to follow-up (n = 26).

Of the 94 individuals assessed for eligibility, 37 were excluded: 9 did not meet fatigue criteria, 15 were not eligible cancer survivors, 6 were already practicing Guolin Qigong, and 7 were carers/supporters. An additional 16 eligible individuals declined participation due to time constraints (n = 3), scheduling conflicts (n = 2), distance (n = 2), illness (n = 3), or change of mind (n = 6). A total of 41 participants were randomised.

After randomisation, 3 participants from usual care waitlist control group dropped out before baseline assessment and intervention due to being too sick (n = 1) and scheduling conflict (n = 2) while one (n = 1) dropped-out on Week-12.

Retention over 12 and 16 weeks of 95% (20/21) for GQ group and 80% (16/20) for the waitlist control group met our priori retention rate of 80% to 85%.^30^

#### Adherence: Class Attendance and Home Practice

The average class attendance rate was 86% (ie, 12 out of 14 classes). This met our priori class attendance rate of 75% to 85% for success.^30^ The average home practice was 5.15 days/week and a mean of 394.5 minutes/week of combined practice for the 3 methods (details of each method in Supplemental Material 1) for the intervention group.

#### Safety

A total of 44 adverse events were recorded of which 42 were Grade 1 events and 1 each of Grade 4 and Grade 5 events. The most common Grade 1 events were musculoskeletal and connective tissue disorders (n = 14), nervous system disorders (n = 8), general disorders and administration site condition (n = 8), and skin and subcutaneous tissue disorders (n = 6). Of the 42 Grade 1 events, 18 (42%) were considered unrelated and 24 (58%) were reported as GQ related. Most of the unrelated events were musculoskeletal aches/pain and fatigue while GQ related adverse events were mainly musculoskeletal aches and pains, paraesthesia from nervous system disorder, and sweating. Please refer to *Supplemental Material 2* for detailed breakdown.

There was a case of death (Grade 5) and one surgery (Grade 4), both unrelated to the study. The death was due to a stage 4 breast cancer that had metastasised to the liver and bones. The participant passed away midway through the intervention period from the progression of liver metastases, which did not respond to treatment. The surgery involved a pre-planned procedure to remove a pre-existing lump in the throat of another participant.

#### Completion of Data Collection

Overall, 20 of 21 (95%) GQ participants and 16 of 20 (80%) of waitlist control participants completed the 12-week protocol and data collection. Missing data were mainly due to one GQ intervention participant who died half way through the 12-week intervention while 3 from the waitlist control group withdrew prior to commencement of the intervention for reasons of being too sick (n = 1) and conflicts of time (n = 2). Another waitlist control participant dropped out in Week 12 without giving any reason (n = 1).

### Secondary Outcomes Results– Preliminary Effects

In Supplemental Material 3, the participant mean scores for fatigue, sleep, and depression are presented, along with the differences between the intervention and control groups from baseline to Week 16. The t-tests did not show significant differences between the intervention and control groups for BFI, CES-D, and PSQI. However, the intervention group showed benefits in certain PSQI subscales, such as subjective sleep quality and latency. Specifically, among cancer participants, there was a statistically significant decrease in the intervention group at Week-12 for subjective sleep quality and sleep latency, with the mean between-group difference (δ) = −0.49, 95% CI = −0.86 to −0.11 for subjective sleep quality and δ = −0.76, 95% CI = −1.35 to −0.17 for sleep latency.

**Figure 2.**
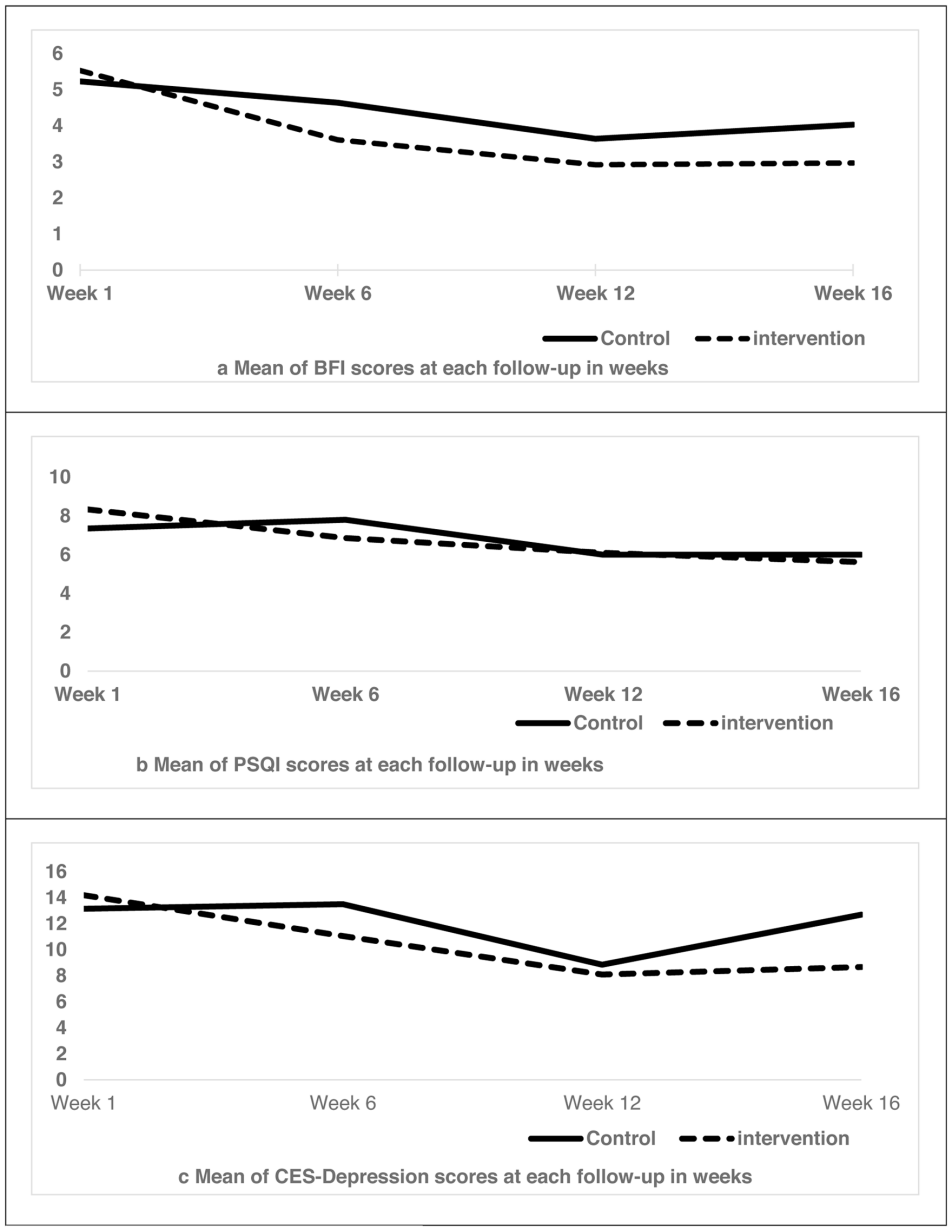
(a-c) mean differences for BFI, CES depression, and PSQI at each follow-up week. Abbreviations: BFI: brief fatigue index; PSQI: Pittsburgh sleep quality index; CES-D: centre for epidemiologic studies depression.

Figure 2a to c compares the intervention and control groups for the follow-up from Week 1 to Week 16. Figure 2a indicates that the intervention group demonstrated benefits in certain areas from Week 6 to Week 16 for BFI. Additionally, intervention benefits were observed in Weeks 6 and 16 for PSQI and CES-D (see Figure 2b and c), but there was a non-statistical reduction in the intervention group.

Table 2 presents the unadjusted and adjusted coefficients for fatigue, sleep, and depression in co-occurring symptoms in survivors over the 16 weeks after adjusting for gender and type of cancer.

**Table 2.** Fatigue, Sleep, and Depression in Co-Occurring Symptoms in Survivors for the 16 Weeks of Follow-Up Visits.

Assessment over 16 wk	Control vs. intervention			Control vs. intervention^a^		
	Coefficient (β)	**95% CI**		β^1^	**95% CI**	
Brief fatigue inventory (BFI)	−.63	−1.49	0.23	−.66	−1.51	0.19
Pittsburgh sleep quality index (PSQI)	−.06	−1.89	1.77	−.11	−1.95	1.72
CES-depression	−1.55	−7.30	4.2	−1.79	−7.66	4.08
Subscale of PSQI						
C1 subjective sleep quality	−.27	−0.57	0.03	−.28	−0.57	0.01
C2 sleep latency	−.33	−0.83	0.17	−.29	−0.79	0.21
C3 sleep duration	−.16	−0.70	0.38	−.25	−0.78	0.27
C4 habitual sleep efficiency	.10	−0.13	0.34	.13	−0.10	0.36
C5 sleep disturbances	.05	−0.22	0.32	.05	−0.22	0.32
C6 use of sleep medication	.29	−0.24	0.81	.25	−0.28	0.77
C7 daytime dysfunction	.16	−0.19	0.51	.17	−0.18	0.52

β^1^, the effect of the intervention in pooled cancer data for 16 weeks using a generalised estimation equations Gaussian family with robust variance estimation to adjust for within-person correlation from repeated follow-up measurements.

a Adjusted for gender and type of cancer.

The results showed no significant differences between the unadjusted and adjusted analyses for both groups. BFI PQSI, and CES-Depression decreased by an average of 0.66, 0.11, and 1.79 respectively in the GQ intervention group. The GQ intervention group also experienced an average decrease of 0.28 and 0.29 in the Subjective Sleep Quality and Sleep Latency subscales of PSQI, respectively.

**Figure 3.**
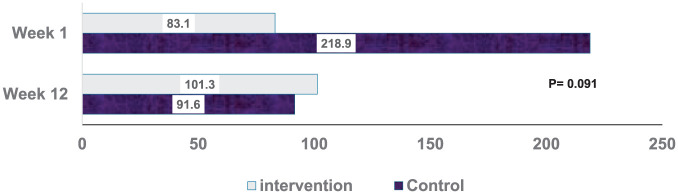
Twenty-four-hour urine cortisol at week-1 and week-12.

Figure 3 shows the mean scores of the 24-hour urine cortisol of the GQ intervention and waitlist control groups on Week 1 and Week 12. Exploratory analysis showed no statistical difference in the 24-hour urine cortisol between the 2 groups before and after intervention.

## Discussion

This study supports the feasibility and safety of GQ as a supportive method for cancer survivors, especially for those with limited options from standard care. It provides valuable insights into recruitment strategies, adherence, safety, and data collection procedures, thereby informing the design of a future fully powered trial. While the primary objective was to assess feasibility, the secondary outcomes assessment on fatigue, sleep disturbance, depression, and urinary cortisol were exploratory in nature and were not intended to establish clinical effectiveness.

### Feasibility of Recruitment and Retention of Participants

The study successfully achieved its recruitment and retention feasibility objectives. The high level of recruitment and retention indicated enthusiasm and interest in GQ among the cancer survivors. The recruitment rate of 72% exceeded typical benchmarks reported for Qigong studies in cancer survivors^44,45^ and exercise oncology trials.^58-60^

Additionally, the enrolment rate of 25% outperformed rates of 3% to 11%^43,61,62^ commonly reported in other trials. For instance, this study successfully recruited 41 participants within 10 months, surpassing a comparable pilot study that required 21 months to enrol only 20 participants.^44^ This achievement was largely facilitated by collaboration with established cancer support networks, which provided access to a motivated pool of survivors accounting for 81% of inquiries. Engagement with these community groups, supported by a multidisciplinary team, comprising healthcare professionals from Western and Chinese medicine, and the conduct of the study within University Malaya Medical Centre, a leading teaching hospital, likely enhanced trust and participation.

Retention rates were equally high, with 95% (20/21) in the GQ group and 80% (16/20) in the waitlist control group at Week-12 and Week-16. These figures outperformed other oncology trials of exercise and Qigong, which typically reported retention rates ranging from 86% to 89%^58-60^ and 39% to 67%^43-45^ respectively. For instance, a recent Qigong trial noted dropout rates in control groups up to 4 times higher than those in intervention groups.^63^

### Adherence of GQ Intervention

#### Training Class Attendance

Attendance rates in this study were high, with participants attending 86% of scheduled sessions exceeding rates reported in similar Qigong studies.^43,44,61,62,64,65^ This success could be attributed to the practical and contextual feature of the programme design. Classes were scheduled on weekends from 3 to 5pm, a time that fell conveniently between lunch and dinner for working participants, and between hospital visiting hours, which facilitated access to parking. The GQ intervention was delivered by experienced GQ instructors who were long-term cancer survivors, which may have enhanced credibility, relatability, and participant trust.

Additionally, the supervised group-based format likely fostered social support, engagement, accountability, and peer connexion. This aligns with the Exercise Guidelines for Cancer Survivors, which emphasise the superior effectiveness of supervised programmes compared to unsupervised or home-based interventions.^60,66^ The informal refreshment breaks during the GQ sessions also created opportunities for participants to connect, share experiences, and build a sense of community. Such camaraderie has been shown to significantly enhance adherence and psychological well-being, aligning with evidence that social interaction, whether from peers, family, or instructors, played a pivotal role in exercise programme success^67,68^ as reflected in the high attendance rate in this study.

Lastly, the integration of culturally relevant health education from a Traditional Chinese Medicine (TCM) perspective may have also resonated with the predominantly Asian cohort,^5^ supporting engagement.^12^

#### Home Practice

Participants reported an averaged practice of 5 days per week and 394 minutes per week of home practice indicating strong engagement with GQ intervention. This level of activity exceeded the U.S. Physical Activity Guidelines of 150 to 300 minutes for adults,^69,70^ suggesting that participants were able to incorporate GQ into their regular routines. The substantial variability in frequency and duration (ranging from 1 to 7 days and 65–590 minutes per week) among the participants reflects diverse levels of individual engagement, and highlights the inherent flexibility of GQ, allowing participants to tailor their practice based on personal health conditions, energy levels, and availability.

This findings support the feasibility and acceptability of sustained home practice within this population.

#### Safety

Adverse events were rigorously monitored throughout the study. Of the 44 events reported, 58% (24/44) identified as associated with GQ were mild and included musculoskeletal discomfort, tingling, numbness, and sweating, responses commonly linked to normal physiological adaptations to exercise.^59,71^ All were transient and self-limiting, possibly indicating the body’s natural adjustments to increased physical activity. Moreover, distinguishing these mild effects from cancer or cancer treatment-related symptoms posed a challenge, suggesting that not all events could be solely attributed to Qigong. When Qigong was implicated, the effects were brief, and self-limiting.

The relatively higher rate of reported adverse events compared to prior Qigong or exercise oncology trials^44,72^ likely reflects this study’s comprehensive monitoring, as many previous studies lacked sufficient reporting or analyses, potentially underreporting adverse events.^32,58-60,73,74^

Importantly, no serious GQ-related adverse events were reported, consistent with existing literature on Qigong for cancer survivors.^16,18,32,75^

### Preliminary Estimates of GQ

Although the intervention group showed benefits in certain PSQI subscales, such as subjective sleep quality and latency, these results should be interpreted cautiously. The study was not powered to detect statistically significant between-group differences in fatigue, sleep disturbance, depression symptoms, or 24-hour urine cortisol. As expected in a pilot feasibility study, wide confidence intervals and largely non-significant group differences limit any conclusions regarding therapeutic benefit.

The absence of statistically significant changes across primary symptom measures reflects the design characteristics of feasibility study such as the relatively short 12-week duration and small sample size in the current study. Prior Qigong studies indicated that longer intervention duration, exceeding 24 weeks, may be associated with are more improvements.^76-78^ Additionally, exercise interventions typically require a minimum dose-response rate to demonstrate significant outcomes, and the absence of this specificity regarding the dose-response effects of GQ and Qigong in general on specific endpoints in cancer population highlights a gap in the field of Qigong mind body exercise oncology.^79^ Furthermore, the engagement of waitlist control participants in this study in other cancer support activities, such as cancer support workshops and exercise programmes, may have provided comparable psychological and physical benefits, thus narrowing the measurable differences between the 2 groups. While participation in such activities reflects the real-world context in which cancer survivors seek multiple sources of support, it also presents a challenge in isolating the effects of GQ. Future studies may consider alternative control group designs, such as an active control with structured but non-Qigong-based interventions, to better differentiate outcomes while ensuring that participants continue to receive beneficial support. Such approaches would help refine the understanding of GQ’s unique contributions without compromising cancer survivors’ access to valuable supportive care resources.

### Strength and Limitation

To our knowledge, this is the first feasibility study that explored the preliminary effects of GQ on fatigue, sleep disturbance, depression and cortisol change in breast, lung, and colon cancer survivors. This study demonstrated strong recruitment, retention, and adherence supported by community collaboration, experienced instructors, structured and supervised delivery, providing insights for planning future larger, more rigorous trials.

At the same time, several limitations should be acknowledged. The small sample size limited statistical power to detect differences in secondary outcomes; however, as a feasibility trial, the primary goal was to assess acceptability and potential signals of benefit rather than definitive efficacy. Although heterogeneity in health status, treatment history, and exercise experience may have introduced variability, it also reflects the reality of survivorship populations and enhances ecological validity. The concentration of participants who were relatively high-functioning further constrained generalisability, as did the lack of ethnic diversity, yet the findings still provide important preliminary insights for similar subgroups and can inform more inclusive future trials. Reliance on self-reported outcomes and absence of validated tools for symptom clusters further constrain interpretation of the findings, highlighting the need for developing and applying standardised assessment tools in subsequent investigations. Nevertheless, these limitations are common in early-phase trials and should be viewed in light of the study’s strengths, which included a diverse cancer sample, structured intervention delivery, and independent data verification. Taken together, this study provides important feasibility data to inform the design of a larger, adequately powered RCT.

## Conclusion

This study demonstrated the feasibility of both the GQ intervention and the RCT methodological procedures among breast, lung, and colon cancer survivors. High recruitment and retention rates highlighted the programme’s acceptability and relevance within this sample, with community collaborations playing an important role in supporting participation. The structured and culturally sensitive design of the supervised intervention likely facilitated engagement. These findings provided insights for scaling up to a future adequately powered RCT to evaluate the effectiveness of GQ within cancer survivorship care.

## Supplemental Material

sj-pdf-1-ict-10.1177_15347354261442682 – Supplemental material for “Walk for Life”: A Feasibility Randomised Controlled Trial of Guolin Qigong for Fatigue, Sleep Disturbance and Depression Symptom Cluster in Cancer Survivors Supplemental material, sj-pdf-1-ict-10.1177_15347354261442682 for “Walk for Life”: A Feasibility Randomised Controlled Trial of Guolin Qigong for Fatigue, Sleep Disturbance and Depression Symptom Cluster in Cancer Survivors by Sara L. K. Low, Gwo Fuang Ho, Bingkai Liu, Eng-Siew Koh, Kingsley Agho, Yutong Fei and Xiaoshu Zhu in Integrative Cancer Therapies

sj-pdf-2-ict-10.1177_15347354261442682 – Supplemental material for “Walk for Life”: A Feasibility Randomised Controlled Trial of Guolin Qigong for Fatigue, Sleep Disturbance and Depression Symptom Cluster in Cancer Survivors Supplemental material, sj-pdf-2-ict-10.1177_15347354261442682 for “Walk for Life”: A Feasibility Randomised Controlled Trial of Guolin Qigong for Fatigue, Sleep Disturbance and Depression Symptom Cluster in Cancer Survivors by Sara L. K. Low, Gwo Fuang Ho, Bingkai Liu, Eng-Siew Koh, Kingsley Agho, Yutong Fei and Xiaoshu Zhu in Integrative Cancer Therapies

sj-pdf-3-ict-10.1177_15347354261442682 – Supplemental material for “Walk for Life”: A Feasibility Randomised Controlled Trial of Guolin Qigong for Fatigue, Sleep Disturbance and Depression Symptom Cluster in Cancer Survivors Supplemental material, sj-pdf-3-ict-10.1177_15347354261442682 for “Walk for Life”: A Feasibility Randomised Controlled Trial of Guolin Qigong for Fatigue, Sleep Disturbance and Depression Symptom Cluster in Cancer Survivors by Sara L. K. Low, Gwo Fuang Ho, Bingkai Liu, Eng-Siew Koh, Kingsley Agho, Yutong Fei and Xiaoshu Zhu in Integrative Cancer Therapies
