# Supplementary material for: “Walk for Life”: A Feasibility Randomised Controlled Trial of Guolin Qigong for Fatigue, Sleep Disturbance and Depression Symptom Cluster in Cancer Survivors
Source: Integr Cancer Ther. 2026 May 4;25:15347354261442682. doi: 10.1177/15347354261442682 (PMC13157540; doi:10.1177/15347354261442682)
Supplement: sj-pdf-1-ict-10.1177_15347354261442682 – Supplemental material for “Walk for Life”: A Feasibility Randomised Controlled Trial of Guolin Qigong for Fatigue, Sleep Disturbance and Depression Symptom Cluster in Cancer Survivors [file sj-pdf-1-ict-10.1177_15347354261442682.pdf]

Supplementary Material 1 Home Practice

Mean and Standard deviation home practice and outcome measures for intervention group participants as below:	
Home Practice	Mean(Sd)
Day/Week	5.15 (1.54)
Method 1 (Minutes/Week)	190.5 (92.6)
Method 2 (Minutes/Week)	181.9 (99.3)
Method 3 (minutes/Week)	22.1 (20.9)
3 Methods Combined (Minutes/Week)	394.5 (172.9)
