# Supplementary material for: “Walk for Life”: A Feasibility Randomised Controlled Trial of Guolin Qigong for Fatigue, Sleep Disturbance and Depression Symptom Cluster in Cancer Survivors
Source: Integr Cancer Ther. 2026 May 4;25:15347354261442682. doi: 10.1177/15347354261442682 (PMC13157540; doi:10.1177/15347354261442682)
Supplement: sj-pdf-2-ict-10.1177_15347354261442682 – Supplemental material for “Walk for Life”: A Feasibility Randomised Controlled Trial of Guolin Qigong for Fatigue, Sleep Disturbance and Depression Symptom Cluster in Cancer Survivors [file sj-pdf-2-ict-10.1177_15347354261442682.pdf]

Supplementary Material 2 Adverse Events

Table **3 Adverse Events**				
**Adverse Events**	**Grade**	**Related Events (n)**	**Unrelated Events (n)**	**Total**
Musculoskeletal and connective tissue disorder	1	10	4	14
Nervous system disorder	1	7	1	8
Skin and subcutaneous tissue disorder	1	6	0	6
General disorders and administration site condition	1	0	8	8
Gastrointestinal disorders	1	0	1	1
Vascular disorder	1	0	1	1
Reproductive system and breast	1	0	1	1
Psychiatric disorders	1	0	2	2
Renal and urinary disorders	1	1	0	1
**Sub-Total Grade 1**		24	18	42
Surgery/Hospitalisation	4	0	1	1
Death	5	0	1	1
**Total**		24	20	44
