# Supplementary material for: “Walk for Life”: A Feasibility Randomised Controlled Trial of Guolin Qigong for Fatigue, Sleep Disturbance and Depression Symptom Cluster in Cancer Survivors
Source: Integr Cancer Ther. 2026 May 4;25:15347354261442682. doi: 10.1177/15347354261442682 (PMC13157540; doi:10.1177/15347354261442682)
Supplement: sj-pdf-3-ict-10.1177_15347354261442682 – Supplemental material for “Walk for Life”: A Feasibility Randomised Controlled Trial of Guolin Qigong for Fatigue, Sleep Disturbance and Depression Symptom Cluster in Cancer Survivors [file sj-pdf-3-ict-10.1177_15347354261442682.pdf]

Supplementary Material 3

Cancer survivors' fatigue, depression, and sleep quality score mean by group over time

Variable	Intervention	Control	Control vs Intervention
	mean±SD	mean±SD	mean difference (95% CI)
**Brief Fatigue Inventory (BFI)**			
Over 16 Weeks	3.8 ±0.3	4.4 ±0.3	-0.63 (-1.49, 0.23)
Week 1	5.5 ±0.4	5.2 ±0.3	0.30 (-0.73, 1.33)
Week 6	3.6 ±0.5	4.6 ±0.5	-1.03 (-2.38, 0.32)
Week 12	2.9 ±0.4	3.6 ±0.4	-0.72 (-1.96, 0.52)
Week 16	3.0 ±0.4	4.0 ±0.4	-1.06 (-2.17, 0.26)
**CES-Depression**			
Over 16 Weeks	10.5 ±2.1	12.1 ±2.1	-1.55 (-7.30, 4.20)
Week 1	14.2 ±2.5	13.2 ±2.5	1.04 (-5.83, 7.91)
Week 6	11.0 ±2.1	13.5 ±3.0	-2.45 (-9.62, 4.71)
Week 12	8.1 ±1.9	8.9 ±1.9	-0.75 (-6.13, 4.62)
Week 16	8.7 ±2.3	12.7 ±3.4	-4.03 (-12.11, 4.05)
**Pittsburgh Sleep Quality Index (PSQI)**			
Over 16 Weeks	6.7 ±0.8	6.8 ±0.5	-0.06 (-1.89, 1.77)
Week 1	8.3 ±0.8	7.3 ±0.7	0.98 (-1.10, 3.07)
Week 6	6.8 ±0.9	7.8 ±0.6	-0.94 (-3.11, 1.22)
Week 12	6.1 ±0.9	6.0 ±0.6	0.09 (-2.02, 2.21)
Week 16	5.6 ±0.9	6.0 ±0.6	0.38 (-2.48, 1.71)
**Subscale of PSQI**			
**C1 Subjective Sleep Quality**			
Over 16 Weeks	1.1±0.1	1.4 ±0.1	-0.27 (-0.56, 0.03)
Week 1	1.6±0.2	1.5 ±0.2	0.07 (-0.40, 0.55)
Week 6	1.3±0.2	1.5 ±0.1	- 0.21 (-0.61, 0.19)
Week 12	0.8±0.2	1.3 ±0.1	**- 0.49 (-0.86, -0.11)**
Week 16	0.9±0.2	1.4 ±0.1	**- 0.44 (-0.88,- 0.01)**
**C2 Sleep Latency**			
Over 16 Weeks	1.3±0.2	1.7 ±0.1	- 0.32 (-0.83, 0.17)
Week 1	1.8±0.2	1.6 ±0.2	0.21 (-0.40, 0.82)
Week 6	1.3±0.2	1.7 ±0.2	-0.37 (-0.98, 0.25)
Week 12	1.1±0.2	1.9 ±0.2	**-0.76 (-1.35,- 0.17)**
Week 16	1.1±0.2	1.5 ±0.2	-0.40 (-0.98, 0.17)
**C3 Sleep Duration**			
Over 16 Weeks	1.0±0.2	1.3 ±0.2	- 0.16 (-0.70, 0.38)
Week 1	1.1±0.2	1.4 ±0.3	- 0.25 (-0.92, 0.41)

Week 6	0.9±0.2	1.4 ±0.3	- 0.44 (-1.11, 0.22)
Week 12	1.1±0.2	1.0 ±0.3	0.10 (-0.57, 0.77)
Week 16	0.8±0.2	0.9 ±0.3	- 0.04 (-0.71, 0.62)
**C4 Habitual Sleep Efficiency**			
Over 16 Weeks	0.2±0.1	0.1 ±0.1	0.10 (-0.13, 0.34)
Week 1	0.2±0.1	0.3 ±0.2	- 0.11 (-0.48, 0.27)
Week 6	0.2±0.1	0.2 ±0.1	0.09 (-0.25, 0.43)
Week 12	0.3±0.1	0.1 ±0.05	0.28 (-0.04, 0.61)
Week 16	0.2±0.1	0.0 ±0.0	0.14 (-0.13, 0.42)
**C5 Sleep Disturbances**			
Over 16 Weeks	1.5±0.1	1.4 ±0.1	0.05 (-0.22, 0.32)
Week 1	1.8±0.1	1.4 ±0.2	0.36 (-0.03, 0.76)
Week 6	1.5±0.1	1.7 ±0.1	- 0.17 (-0.48, 0.13)
Week 12	1.4±0.1	1.3 ±0.1	0.08 (-0.30, 0.49)
Week 16	1.2±0.1	1.3 ±0.1	- 0.06 (-0.40, 0.27)
**C6 Use of Sleep Medication**			
Over 16 Weeks	0.5±0.2	0.3 ±0.1	0.29 (-0.24, 0.81)
Week 1	0.6±0.3	0.2 ±0.1	0.41 (-0.16, 0.99)
Week 6	0.6±0.2	0.4 ±0.2	0.17 (-0.43, 0.78)
Week 12	0.6±0.2	0.3 ±0.2	0.27 (-0.32, 0.87)
Week 16	0.4±0.2	0.2 ±0.1	0.27 (-0.26, 0.82)
**C7 daytime Dysfunction**			
Over 16 Weeks	1.0±0.1	0.9 ±0.2	0.16 (-0.19, 0.51)
Week 1	1.3±0.1	1.1 ±0.1	0.28 (-0.10, 0.66)
Week 6	1.0±0.2	1.1 ±0.1	0.00 (-0.45, 0.45)
Week 12	0.9±0.1	0.8 ±0.2	0.06 (-0.40, 0.51)
Week 16	0.9±0.2	0.7 ±0.2	0.30 (-0.19, 0.79)
